# GLP-1 agonists for smoking cessation and post-cessation weight management: A systematic review and meta-analysis of randomized trials

**DOI:** 10.18332/tid/219815

**Published:** 2026-07-26

**Authors:** Javad Heshmati, Salma Mahmoodianfard, Ethan Raizman, Aidan Abraham, Hassan Mir

**Affiliations:** 1 University of Ottawa Heart Institute, Ottawa, Canada; 2 Ottawa Model for Smoking Cessation, Ottawa, Canada; 3 Faculty of Medicine, University of Ottawa, Ottawa, Canada

**Keywords:** GLP-1 receptor agonists, smoking cessation, abstinence, weight, BMI

## Abstract

**INTRODUCTION:**

Smoking and obesity are leading risk factors for morbidity and mortality worldwide. Recent studies have demonstrated the efficacy of glucagon-like peptide-1 (GLP-1) receptor agonists in reducing weight and improving metabolic outcomes. Emerging evidence suggests that these agents may also influence reward-related pathways, offering potential benefit for nicotine addiction and mitigating post-cessation weight gain. This study aimed to evaluate the efficacy of GLP-1 receptor agonists for smoking cessation and their effects on post-cessation weight and BMI.

**METHODS:**

A systematic review and meta-analysis of randomized controlled trials was conducted, with the literature searched through November 2025 in PubMed, Scopus, the Cochrane Library, and Web of Science. Eligible studies included adult smokers (Population) receiving GLP-1 receptor agonists (Intervention) compared with placebo or standard care (Comparator), reporting smoking cessation and/ or post-cessation weight or BMI outcomes (Outcomes). Random-effects models were used to pool data, with odds ratios (ORs) for abstinence and weighted mean differences (WMDs) for weight and BMI, and heterogeneity assessed using the I^2^ statistic.

**RESULTS:**

Of the 751 records screened, 26 full texts were reviewed, and two randomized controlled trials (n=337) were included. Compared with placebo, GLP-1 receptor agonists showed no significant effect on smoking cessation (OR=1.36; 95% CI: 0.55–3.35, p=0.51; I^2^=66.93%; very low certainty), but significantly reduced post-cessation body weight (WMD=4.14 kg, 95% CI: -7.22 – -1.05, p=0.01; I^2^=19.93%; low certainty) and BMI (WMD= -1.77 kg/m^2^, 95% CI: -2.20 – -1.34, p=0.00; I^2^=00.0%; low certainty).

**CONCLUSIONS:**

While GLP-1 receptor agonists have revolutionized the treatment of diabetes and obesity, their role in smoking cessation remains inconclusive. However, reductions in post-cessation weight and BMI highlight their potential as adjunctive agents to address weight-related relapse risk. Larger, longer duration trials with newer GLP-1 agents are warranted to confirm these findings and clarify their role in smoking cessation.

## INTRODUCTION

Smoking is a leading cause of global morbidity and mortality^1^. An estimated 1.3 billion people worldwide use tobacco products, 80% of whom are in low- and middle-income countries^2,3^. Smoking is most prevalent in adults aged 25–44 years, and the uptake of newer products such as e-cigarettes continues to rise in this demographic^4^. In 2019, smoking was responsible for ~7.7 million deaths and 200 million disability-adjusted life years (DALYs) globally^5^.

While smoking cessation can mitigate or reverse certain consequences of smoking, relapse rates are high^6^. Approximately 75% of smokers relapse within 6 months^7^. Behavioral counseling, nicotine replacement therapy (NRT), bupropion, and varenicline are established smoking cessation interventions^8,9^. Among these, bupropion is a norepinephrine-dopamine reuptake inhibitor, while varenicline is a partial agonist at the α4β2 nicotinic acetylcholine receptor^10^. NRTs are widely available in forms such as patches, gums, and lozenges, typically achieving 6-month abstinence rates of 20% to 30%^11^. Each cessation method comes with limitations: bupropion may increase the risk of seizures in susceptible populations^12^, varenicline can cause sleep disturbances and vivid dreams^13^, and NRT often leads to weight gain and may not adequately address the psychological dependence associated with smoking.

A major barrier to long-term smoking abstinence is nicotine dependence itself, along with physiological and behavioral withdrawal symptoms^14^. Post-cessation weight gain is a frequent and distressing withdrawal symptom that can discourage quit attempts and increase relapse risk^15^. Nicotine activates central and peripheral nicotinic acetylcholine receptors (nAChRs), which modulate both homeostatic feeding pathways and reward-related circuits involved in appetite and food intake^16^. Understanding how these pathways interact provides a rationale for exploring pharmacotherapies that can simultaneously target addiction and metabolism.

Glucagon-like peptide-1 (GLP-1) receptor agonists are established treatments for type 2 diabetes and obesity, demonstrating significant benefits in glycemic control, weight reduction, and cardiometabolic outcomes^17^. Their use has expanded rapidly, with 12% of US adults having ever used and 6% currently using one^18,19^. Beyond their cardiometabolic effects, GLP-1 has been shown to improve clinical outcomes such as Major Adverse Cardiovascular Events (MACE) and Obstructive Sleep Apnea (OSA)^20,21^. Emerging evidence also implicates GLP-1 signaling in reward and addiction pathways^22^. Preclinical data suggest that GLP-1 receptor agonists suppress nicotine-induced dopamine release in mesolimbic regions, thereby reducing the reinforcing effects of smoking^23^. Additionally, these agents stimulate insulin secretion and reduce energy intake, which may help mitigate post-cessation weight gain^19,24^. Given this dual effect on both addiction-related neurocircuitry and metabolic regulation, evaluating outcomes such as body weight and BMI alongside abstinence provides a more comprehensive understanding of the therapeutic potential of GLP-1 receptor agonists in smoking cessation.

Preliminary human studies support this rationale. Trials of exenatide and dulaglutide have demonstrated trends toward higher abstinence rates and significant reductions in post-cessation weight and BMI^25^. However, while GLP-1 receptor agonists have been extensively studied for their effects on weight management, their impact on smoking cessation has only been investigated in a limited number of trials^26^. While several narrative reviews have discussed GLP-1 receptor agonists in substance use disorders broadly^27-33^, no prior systematic review has specifically focused on randomized controlled trials (RCT) evaluating their effects on smoking cessation.

Synthesizing the research to date is important for several reasons. First, clinicians and researchers are increasingly exploring the therapeutic repurposing of GLP-1 receptor agonists beyond metabolic disease, including their application in addiction medicine. Second, ongoing trials listed on ClinicalTrials. gov^34^ vary substantially in design, populations, and outcomes, highlighting the need for early evidence mapping to inform future research directions and trial methodology. Third, post-cessation weight gain remains one of the strongest predictors of relapse^35^, and understanding whether GLP-1 receptor agonists confer metabolic protection during cessation may inform tailored interventions. Finally, given the global burden of smoking and the limited success of existing cessation therapy, early signals of efficacy, even from small RCTs, are clinically relevant.

This systematic review and meta-analysis evaluate the efficacy of GLP-1 receptor agonists for smoking cessation and their effect on post-cessation weight and BMI. By synthesizing available evidence, this study aims to identify research gaps, inform clinical practice, and guide the design of future trials exploring GLP-1 receptor agonists as dual-action therapies for smoking cessation and weight management.

## METHODS

The current systematic review and meta-analysis aimed to evaluate the efficacy of GLP-1 receptor agonists as a smoking cessation aid and their impact on post-cessation weight and BMI changes. This review followed a prospectively registered protocol (PROSPERO CRD42024567807) and adhered to PRISMA (Preferred Reporting Items for Systematic Reviews and Meta-Analyses) guidelines to ensure comprehensive reporting standards and transparency.

### Search strategy

A comprehensive electronic search was performed in PubMed, Scopus, the Cochrane Library, and Web of Science from database inception to 5 November 2025. The search was conducted using predefined terms (detailed in Supplementary file Material 1) and imposed no language or geographical restrictions. In addition to the systematic searches, bibliographies of identified studies and relevant review articles were examined to uncover additional randomized controlled trials (RCTs) that met the eligibility criteria.

### Eligibility criteria

Studies were eligible if they adhered to specific criteria: 1) participants included people who smoke, vape, or those with nicotine dependence; 2) interventions compared GLP-1 receptor agonists or antagonists to placebo or no intervention; and 3) reported smoking or vaping abstinence as a primary or secondary outcome. Secondary outcomes included changes in body weight and BMI following the intervention to evaluate whether GLP-1 receptor agonists influence post-cessation metabolic outcomes. Only randomized placebo-controlled trials were included, while review articles, observational studies, and brief reports were excluded. Two reviewers (E.R. and A.A) screened all titles and abstracts in duplicate, resolving any disagreements through discussions with additional team members (J.H. and H.M.). Full-text screening was then performed in duplicate using the same process.

### Data extraction and quality evaluation

Two authors (J.H. and E.R.) independently extracted data using a predefined template and assessed risk of bias using the Cochrane Risk of Bias 2 (ROB 2) tool for randomized trials^36^. The extracted data encompassed study design characteristics, participant details such as age and BMI, and treatment specifics, including the type and dose of GLP-1 receptor agonist. For each included trial, data were extracted for both smoking cessation (abstinence rates and odds ratios) and metabolic parameters (mean and standard deviation of body weight and BMI before and after the intervention).

Any disagreements were resolved through discussion or by consulting a third reviewer (H.M.). The overall risk of bias was classified into low, some concerns, or high based on the evaluation of key domains. The Grading of Recommendations Assessment, Development, and Evaluation (GRADE) approach was conducted to evaluate the quality and certainty of the evidence for the results^37^.

### Data analysis

The meta-analysis was conducted using STATA version 17.0 (STATA Corporation, College Station, Texas, USA)^38^, with pooled odds ratios (ORs) and 95% confidence intervals (CIs) calculated using random-effects models with the REML method. Statistical heterogeneity was evaluated using Q tests and the I^2^ statistic. Sensitivity analyses were performed by systematically excluding eligible trials and studies involving albiglutide. For continuous outcomes (weight and BMI), weighted mean differences (WMDs) with 95% CIs were calculated using the same random-effects model. Data were harmonized by converting weight to kilograms and using a correlation coefficient (r=0.8) to estimate the standard deviation (SD) of change when not reported. The primary outcome was the overall point prevalence of smoking abstinence when comparing the effects of GLP-1 receptor agonists (RA) with placebo, using two-tailed tests and a significance threshold of p<0.05. Secondary outcomes included changes in body weight (kg) and BMI (kg/m^2^) following intervention. Forest plots were generated to display pooled effects for abstinence, weight, and BMI.

## RESULTS

**Figure 1:**
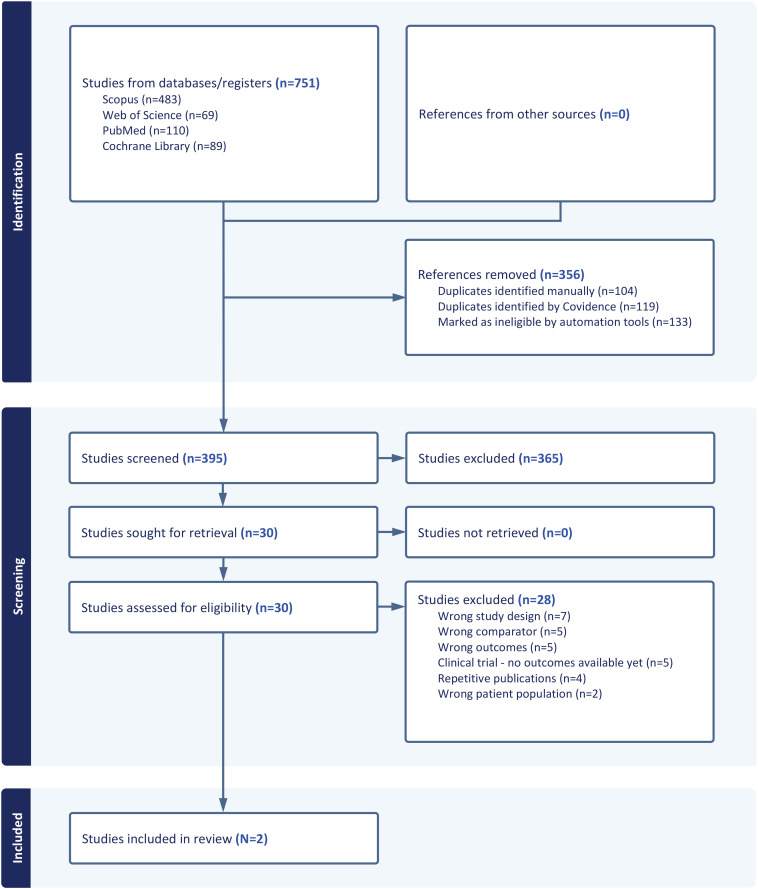
PRISMA flow diagram of study selection for the systematic review and meta-analysis of randomized controlled trials evaluating GLP-1 receptor agonists for smoking cessation and post-cessation weight outcomes (N=2; literature search up to November 2025)

Of the original 751 records, 30 full-text articles were assessed for eligibility. Twenty-eight studies were excluded for several reasons, including study design (7), outcome (5), incomplete (5), duplicate publications (4), incorrect population (2), and inappropriate comparator (5). Finally, two randomized clinical trials^39,40^ were included in this meta-analysis. The PRISMA flow diagram of study selection is presented in Figure 1.

### Description of studies

Main characteristics of included studies are presented in Table 1. Overall, 337 participants were included in this meta-analysis. The Lengsfeld et al.^39^ study involved 255 current smokers (127 intervention, 128 control), and the Yammine et al.^40^ study included 84 prediabetic or overweight people who smoked (42 intervention, 42 control).

**Table 1:** Characteristics of the randomized controlled trials included in the meta-analysis evaluating GLP-1 receptor agonists for smoking cessation and post-cessation weight outcomes (N=2; literature search up to November 2025)

*Authors Year*	*Population*	*Intervention/* *comparator*	*Duration*	*Dose of the* *drugs*	*Age of* *participants*	*BMI of* *participants*	*Main outcomes*
Lengsfeld et al.^39^ 2023	255 participants, 127 participants in experimental group with dulaglutide Eligibility criteria: - Aged 18–75 years - Daily smokers with a minimum of a moderate cigarette dependence (Fagerström score of ≥5 points)	Intervention: Dulaglutide weekly subcutaneous injection + varenicline and behavioral counselling (n=127) Comparator: Placebo injection + varenicline and behavioral counselling (n=128)	12 weeks	First dose (week 1): 0.75 mg/0.5 mL Following doses (weeks 2–12): 1.5 mg/0.5 mL	Overall: 43.2 (SD=13.1) Dulaglutide group: 42.7 (SD=13.8) Control: 43.2 (SD=13.1)	Overall: 27.1 (SD=5.0) Dulaglutide group: 27.1 (SD=5.1) Control: 27.1 (SD=5.0)	63% in the dulaglutide group quit smoking, while 65% in the control group quit smoking – no significant difference between the two groups A weight reduction of -1 kg (SD=2.7) was seen in the dulaglutide group, while a weight gain of 1.9 kg (SD=2.4) was seen in the control group Dulaglutide effectively reduced post-cessation weight gain and had a positive impact on glucose metabolism (decreased HbA1c levels) but not for cravings
Yammine et al.^40^ 2021	82 participants, prediabetic or overweight smokers Eligibility criteria: - Males/females aged 18–75 years - Smoking for minimum 1 year and currently smoking over 10 cigarettes/day - Want to quit smoking - 5.7–6.4% glycosylated hemoglobin levels and/or BMI ≥25 kg/m^2^	Intervention: Extended-release exenatide (2 mg weekly) + nicotine patch (21 mg) and counseling (n=41) Comparator: Placebo injection + nicotine patch (21 mg) and counseling (n=41)	6 weeks	2 mg	Total sample: 51.1 (SD=9.2) Exenatide group: 51.0 (SD=9.1) Placebo group: 51.2 (SD=9.4)	All participants had a BMI of ≥25 kg/m^2^ (or 5.7–6.4% glycosylated hemoglobin levels)	Exenatide treatment with patches showed greater abstinence, decreased cravings, decreased withdrawal symptoms and reduced post-cessation weight gain compared to the patches alone (placebo) Abstinence in exenatide group: 46.3% Abstinence in placebo group: 26.8% Post-cessation body weight was 5.6 lbs lower in the exenatide group compared to placebo

In Lengsfeld et al.^39^, dulaglutide was administered subcutaneously weekly for 12 weeks, starting at 0.75 mg in the first week and increasing to 1.5 mg thereafter, in combination with behavioral counseling and varenicline, as per national guidelines. In Yammine et al.^40^, extended-release exenatide (2 mg weekly for 6 weeks) was provided alongside a 21 mg nicotine patch and behavioral counseling. The mean age of participants in the Lengsfeld et al.^39^ study was 43.2 years (SD=13.1), with the dulaglutide group averaging 42.7 years (SD=13.8) and the control group 43.2 years (SD=13.1). In the Yammine et al.^40^ study, the total sample had a mean age of 51.1 years (SD=9.2), with the exenatide group averaging 51.0 years (SD=9.1) and the placebo group 51.2 years (SD=9.4). In the Lengsfeld et al.^39^ study, the baseline BMI of participants was 32.8 ± 4.8 kg/m^2^, whereas in the Yammine et al.^40^ trial, participants had a BMI ≥25 kg/m^2^, with most classified as overweight or obese. Smoking cessation outcomes in both trials were biochemically verified using exhaled carbon monoxide (CO) ≤5 ppm to confirm 7-day point-prevalence abstinence, thereby ensuring objective verification of self-reported abstinence. The Yammine et al.^40^ study conducted analyses using a modified intention-to-treat (mITT) approach, including all randomized participants who received at least one dose of the study medication. In contrast, the Lengsfeld et al.^39^ study employed a full intention-to-treat (ITT) analysis framework, in which all randomized participants were analyzed according to their assigned groups regardless of treatment adherence or protocol deviations.

Main outcomes from the Lengsfeld et al.^39^ study indicated that 63% of participants in the dulaglutide group and 65% in the control group achieved abstinence, showing no significant difference between groups. However, dulaglutide led to a significant reduction in post-cessation body weight (-4.6 ± 3.7 kg) and BMI (-1.6 ± 1.2 kg/m^2^) compared with placebo (0.5 ± 2.3 kg and 0.2 ± 0.8 kg/m^2^, respectively). In the Yammine et al.^40^ study, exenatide treatment combined with nicotine patches resulted in significantly greater smoking abstinence (46.3% vs 26.8%; p<0.05, based on Bayesian posterior probability = 96.5%), decreased cravings, and reduced withdrawal symptoms. Additionally, the exenatide group exhibited lower post-cessation body weight, with an average reduction of approximately 5.6 lbs (-2.5 kg) compared to the placebo group, though BMI remained relatively stable.

### Quality appraisal and certainty of evidence

**Figure 2:**
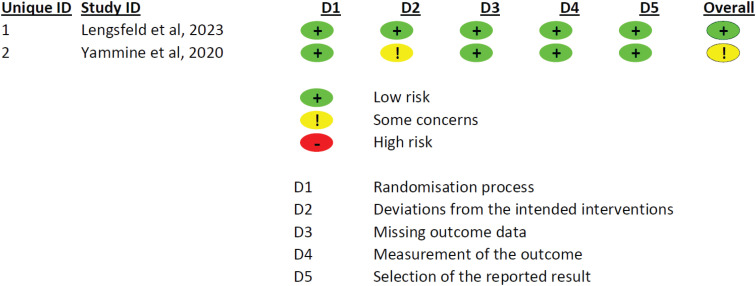
Risk of bias assessment of the randomized controlled trials included in the meta-analysis evaluating GLP-1 receptor agonists for smoking cessation and post-cessation weight outcomes (N=2; literature search up to November 2025), assessed using the Cochrane Risk of Bias 2 (RoB 2) tool

One trial was rated as low risk of bias (low risk of bias across all domains, or low risk of bias in four domains and one domain with ‘some concern’), and the other study was rated as some concern of risk of bias. No major discrepancies arose between reviewers during assessment. A summary of the ROB2 results is shown in Figure 2.

Using the GRADE approach^41^, certainty of evidence for smoking abstinence was rated very low due to serious risk of bias, inconsistency (I^2^ >60%), and imprecision of the pooled estimate. Certainty for body weight and BMI was rated as low, downgraded for risk of bias and imprecision, but not for inconsistency, as both included RCTs demonstrated consistent direction and magnitude of effect favoring GLP-1 receptor agonists. Findings are summarized in Supplementary file Material 2.

### Meta-analysis

**Figure 3:**
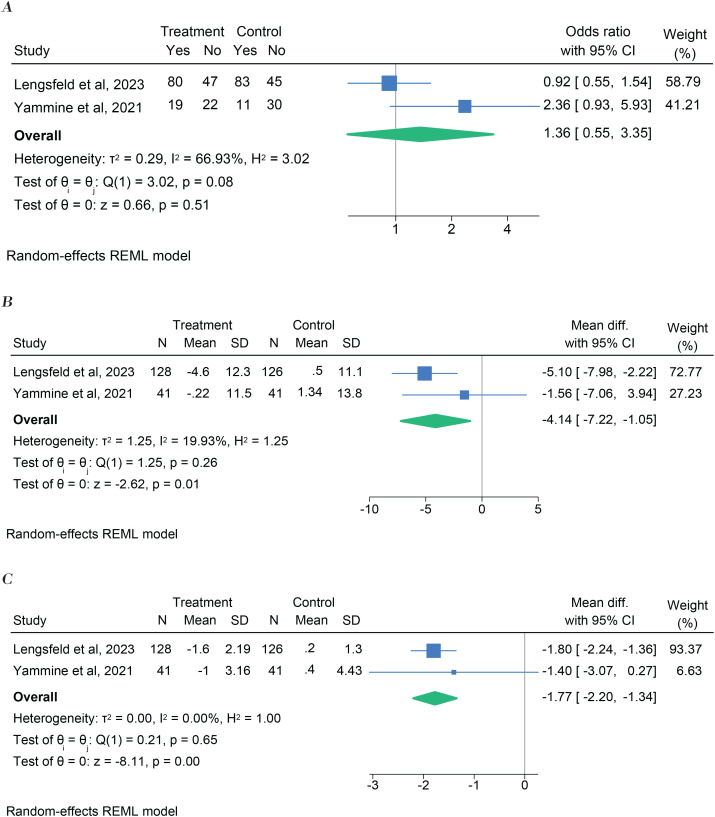
Forest plots of pooled effect estimate from randomized controlled trials evaluating GLP-1 receptor agonists for smoking cessation and post-cessation weight outcomes included in the meta-analysis (N=2; literature search up to November 2025): A) point prevalence abstinence; B) body weight; and C) body mass index (BMI)

A random-effects model meta-analysis of effect estimates from the two included studies indicated that there is no significant effect of GLP-1 receptor agonists on the point prevalence abstinence of smoking cessation (OR=1.36; 95% CI: 0.55–3.35, p=0.51; I^2^=66.9%) (Figure 3A). The high heterogeneity suggests variability in participant characteristics, intervention type, and treatment duration across studies.

For secondary outcomes, GLP-1 receptor agonists produced a statistically significant reduction in post-cessation body weight and BMI. The pooled weighted mean difference (WMD) for weight was -4.14 (95% CI: -7.22 – -1.05, p=0.01; I^2^=19.93%) (Figure 3B), indicating a moderate effect favoring GLP-1 therapy over placebo in limiting post-cessation weight gain. Similarly, the pooled WMD for BMI was -1.77 (95% CI: -2.20 – -1.34, p>0.001; I^2^=00.00%) (Figure 3C).

## DISCUSSION

The efficacy of GLP-1 receptor agonists as a smoking cessation agent is unknown. This systematic review and meta-analysis did not find a significant increase in smoking abstinence with the use of GLP-1 receptor agonists compared with placebo. However, this finding should be interpreted cautiously, as the limited sample size and number of included trials may have resulted in insufficient statistical power to detect a true effect. As expected, GLP-1 receptor agonists were associated with significant reductions in post-cessation body weight and BMI. The observed improvements likely reflect the established pharmacological effects of these agents rather than a smoking cessation–specific benefit. Nonetheless, limiting weight gain during quit attempts is clinically relevant, as weight gain is a common reason for smoking cessation relapse^42,43^. To our knowledge, this is the first systematic review and quantitative synthesis evaluating GLP-1 receptor agonists for smoking cessation.

The use of GLP-1 agonists as a treatment modality for addiction is an area of growing interest. There are several possible mechanisms through which these drugs could influence smoking cessation. Firstly, they modulate the mesolimbic dopamine system, which is involved in the reward and pleasure pathways^44^. By reducing the release of dopamine in response to nicotine, GLP-1 receptor agonists may blunt the rewarding effects of smoking and reduce cravings^45,46^. Additionally, these drugs appear to influence addictive behaviors more broadly, potentially decreasing voluntary nicotine intake and seeking behaviors^47,48^. Furthermore, nicotine activates GLP-1 neurons, and GLP-1 receptor agonists may alter these nicotine-induced effects, making smoking less appealing^49^. Beyond these neurobehavioral mechanisms, GLP-1 receptor agonists also act peripherally to regulate appetite, gastric emptying, and energy intake, which may explain the observed reductions in body weight and BMI during smoking cessation^50,51^. This dual central and metabolic action suggests that GLP-1 therapies could serve as adjunctive interventions not only to attenuate nicotine cravings but also to prevent post-cessation weight gain. Together, these complementary mechanisms support the potential of GLP-1 receptor agonists as multifaceted agents for smoking cessation.

While our meta-analysis did not find a significant increase in abstinence rates with GLP-1 receptor agonists, the available evidence remains limited and inconclusive regarding their role in smoking cessation. It is possible that these short-term interventions are insufficient to sustain abstinence, emphasizing the need for long-term strategies to reduce relapse. Prior studies have shown that extended behavioral and pharmacological support remains critical, as relapse rates remain high despite continued counseling^52^. A recent meta-analysis of 13 RCTs indicated that longer treatment durations (>12 weeks) of pharmacotherapies like varenicline improve relapse prevention^53^. Future research should explore extended GLP-1 therapy in combination with established cessation supports to optimize both abstinence and metabolic outcomes.

Given their established role in weight management^54,55^, a GLP-1 receptor agonist may address one of the most distressing challenges of smoking cessation – post-cessation weight gain^56-58^. In our included studies, GLP-1 receptor agonists were associated with reductions in post-cessation weight and BMI compared with placebo, underscoring their potential value as adjunctive agents that target metabolic barriers to abstinence. These findings indicate the potential for combining GLP-1 agonists with current therapies such as behavioral counseling, cytisine, NRT, or varenicline. Although our pooled analysis did not show a direct improvement in smoking abstinence, the metabolic benefits observed suggest that GLP-1 therapy could indirectly support long-term cessation by reducing the relapse risk associated with weight gain. Such an integrative approach may improve both physical and psychological outcomes during the cessation process.

### Limitations

This review has several limitations, including the small number of published studies, limited sample sizes, and heterogeneity in study design, treatment duration, and co-interventions. The small number of included trials also limits the statistical power of the pooled analysis.

As such, the results should be interpreted cautiously, as a true treatment effect cannot be excluded. In addition, publication bias could not be formally assessed due to the very small number of included studies. With only two trials available, methods such as funnel plots or statistical tests for small-study effects are not informative. Furthermore, the most commonly used agents for diabetes and weight loss, semaglutide and tirzepatide, have not yet been assessed for this indication. Both included RCTs examined GLP-1 receptor agonists as adjuncts rather than as stand-alone therapies, restricting assessment of their independent effects on abstinence. Although both included trials showed consistent directionality of effect for weight and BMI outcomes, heterogeneity in abstinence results underscores the need for standardized trial designs and harmonized outcome reporting.

In summary, GLP-1 receptor agonists appear to offer metabolic benefits during smoking cessation, but their direct effect on abstinence remains unproven. Future large, long-duration RCTs using standardized cessation protocols should evaluate newer GLP-1 agents and explore their integration with behavioral and pharmacological therapies to optimize smoking cessation and metabolic outcomes.

## CONCLUSIONS

This systematic review and meta-analysis provides early quantitative evidence on the potential adjunctive role of GLP-1 receptor agonists in smoking cessation. Across the two included randomized controlled trials, GLP-1 receptor agonists did not significantly improve abstinence compared with placebo. However, they were associated with reductions in post-cessation body weight and BMI, highlighting their well-established weight-loss properties. While this effect may still be clinically relevant in reducing concerns about post-cessation weight gain, the results should be interpreted with caution, given the small number of available studies and the low-to-very-low certainty of evidence. Large, long-term randomized controlled trials are warranted to clarify whether GLP-1 receptor agonists can simultaneously support smoking cessation and promote favorable metabolic outcomes.

## Supplementary Material



## Data Availability

The data supporting this research are available from the authors on reasonable request.
