# Supplementary material for: GLP-1 agonists for smoking cessation and post-cessation weight management: A systematic review and meta-analysis of randomized trials
Source: Tob Induc Dis. 2026 Jul 26;24:10.18332/tid/219815. doi: 10.18332/tid/219815 (PMC13417961; doi:10.18332/tid/219815)
Supplement: Supplementary file 1 [file TID-24-128-s1.pdf]

TID
Tobacco Induced Diseases

Supplementary file

© 2026 Heshmati J. et al.

DOI:
10.18332/tid/219815

The content has been provided by the author(s) and has not been reviewed, verified, or endorsed by European Publishing. It may not have undergone peer review. The views, opinions, and recommendations expressed are solely those of the author(s) and do not necessarily reflect the position of European Publishing. European Publishing accepts no responsibility or liability for any consequences arising from the use of, or reliance on, this content.

Supplementary file Material 1

Adjunctive Use of GLP-1 Agonists for Smoking Cessation and Post-Cessation Weight Management: A Systematic Review and Meta-Analysis of Randomized Trials

Groups	Descriptors
Outcome	“Smoking” OR “Smoking Cessation” OR “Vaping” OR “Nicotine” OR “Tobacco” OR “Nicotine Dependency” OR “Nicotine Addiction” OR “Smoking Quit” OR “Tobacco Cessation” OR “Vaping Cessation” OR “Smoking Abstinence” OR "Nicotine Use Disorder" OR "Nicotine Use Disorder (NUD)" OR "Cigarettes" OR "Cannabis [smoking]" OR "Cigarette smoking"
Exposure	"Glucagon-like peptide-1 (GLP-1) receptor agonists" OR "GLP-1 Analogues" OR "Liraglutide" OR "Exenatide" OR "Dulaglutide" OR "Semaglutide"

PUBMED

Number of localized studies: 110
Limits: -
Number of studies after applying limits: 110

	Descriptors	Number of studies reached
#1	"Smoking"[MeSH Terms] OR "Smoking"[All Fields] OR "Smoking Cessation"[MeSH Terms] OR "Smoking Cessation"[All Fields] OR "Vaping"[MeSH Terms] OR "Vaping"[All Fields] OR "Nicotine"[MeSH Terms] OR "Nicotine"[All Fields] OR "Tobacco"[All Fields] OR "Nicotine Dependency"[All Fields] OR "Nicotine Addiction"[All Fields] OR "Smoking Quit"[All Fields] OR "Tobacco Cessation"[All Fields] OR "Vaping Cessation"[All Fields] OR "Smoking Abstinence"[All Fields] OR "Nicotine Use Disorder"[All Fields] OR "Cigarettes"[All Fields] OR "cannabis smoking"[All Fields] OR "Cigarette smoking"[All Fields]	472726
#2	"glucagon like peptide 1 glp 1 receptor agonists"[All Fields] OR "GLP-1 Analogues"[All Fields] OR "Liraglutide"[MeSH Terms] OR "Liraglutide"[All Fields] OR "Exenatide"[MeSH Terms] OR "Exenatide"[All Fields] OR "Dulaglutide"[All Fields] OR "Semaglutide"[All Fields]	12674
#3	#1 AND #2	110

WEB OF SCIENCE

Number of localized studies: 69
Limits: documents types (articles)
Number of studies after applying limits: 69

	**Descriptors**	Number of studies reached
**#1**	TS=("Smoking") OR TS=("Smoking Cessation") OR TS=("Vaping") OR TS=("Nicotine") OR TS=("Tobacco") OR TS=("Nicotine Dependency") OR TS=("Nicotine Addiction") OR TS=("Smoking Quit") OR TS=("Tobacco Cessation") OR TS=("Vaping Cessation") OR TS=("Smoking Abstinence") OR TS=("Nicotine Use Disorder") OR TS=("Nicotine Use Disorder (NUD)") OR TS=("Cigarettes") OR TS=("Cannabis [smoking]") OR TS=("Cigarette smoking")	496079
**#2**	TS=("Glucagon-like peptide-1 (GLP-1) receptor agonists") OR TS=("GLP-1 Analogues") OR TS=("Liraglutide") OR TS=("Exenatide") OR TS=("Dulaglutide") OR TS=("Semaglutide")	13919
**#4**	**#1** AND **#2** AND **#3**	69

SCOPUS

Number of localized studies: 483
Limits: -
Number of studies after applying limits: 483

	**Descriptors**	Number of studies reached
**#1**	TITLE-ABS-KEY ("Smoking" OR "Smoking Cessation" OR "Vaping" OR "Nicotine" OR "Tobacco" OR "Nicotine Dependency" OR "Nicotine Addiction" OR "Smoking Quit" OR "Tobacco Cessation" OR "Vaping Cessation" OR "Smoking Abstinence" OR "Nicotine Use Disorder" OR "Cigarettes" OR "Cigarette Smoking" OR "Cannabis Smoking")	758836
**#2**	(TITLE-ABS-KEY ("Glucagon-like peptide-1 (GLP-1) receptor agonists")) OR (TITLE-ABS-KEY ("GLP-1 Analogues")) OR (TITLE-ABS-KEY ("Liraglutide")) OR (TITLE-ABS-KEY ("Exenatide")) OR (TITLE-ABS-KEY ("Dulaglutide")) OR (TITLE-ABS-KEY ("Semaglutide"))	22285
**#3**	**#1** AND **#2** AND	483

COCHRANE

Number of localized studies: 89
Limits: trials
Number of studies after applying limits: 89

	**Descriptors**	Number of studies reached
**#1**	("Smoking"):ti,ab,kw OR ("Smoking Cessation"):ti,ab,kw OR ("Vaping"):ti,ab,kw OR ("Nicotine"):ti,ab,kw OR ("Tobacco"):ti,ab,kw OR ("Nicotine Dependency"):ti,ab,kw OR ("Nicotine Addiction"):ti,ab,kw OR ("Smoking Quit"):ti,ab,kw OR ("Tobacco Cessation"):ti,ab,kw OR ("Vaping Cessation"):ti,ab,kw OR ("Smoking Abstinence"):ti,ab,kw OR ("Nicotine Use Disorder"):ti,ab,kw OR ("Nicotine Use Disorder (NUD)"):ti,ab,kw OR ("Cigarettes"):ti,ab,kw OR ("Cannabis [smoking]"):ti,ab,kw OR ("Cigarette smoking"):ti,ab,kw	47695
**#2**	("Glucagon-like peptide-1 (GLP-1) receptor agonists"):ti,ab,kw OR ("GLP-1 Analogues"):ti,ab,kw OR ("Liraglutide"):ti,ab,kw OR ("Exenatide"):ti,ab,kw OR ("Dulaglutide"):ti,ab,kw OR ("Semaglutide"):ti,ab,kw	5792
**#3**	**#1** AND **#2**	89

Supplementary file Material 2

GRADE certainty of evidence

Certainty assessment							№ of patients		Effect		Certainty	Importance
№ of studies	Study design	Risk of bias	Inconsistency	Indirectness	Imprecision	Other considerations	GLP-1 Agonists	placebo	Relative (95% CI)	Absolute (95% CI)		
**Point Prevalence Abstinence**												
2	randomised trials	serious[a]	serious[b]	not serious[c]	very serious[c,d]	none	99/168 (58.9%)	94/169 (55.6%)	not estimable	**1 more per 1,000** (from 1 fewer to 3 more)	⊕◯◯◯ Very low[a,b,c,d]	IMPORTANT
**Weight**												
2	randomised trials	serious[a]	not serious	not serious[c]	serious[c]	none	169	167	-	-	⊕⊕◯◯ Low[a,c]	IMPORTANT
**BMI**												
2	randomised trials	serious[a]	not serious	not serious[c]	serious[c]	none	169	167	-	-	⊕⊕◯◯ Low[a,c]	IMPORTANT

CI: confidence interval, **BMI**: Body Mass Index, **SMD:** standardised mean difference

Explanations

a. The quality of the evidence was downgraded due to concerns about the risk of bias in the included studies.

b. Downgraded since I2 of heterogeneity of included studies are more than 60%

c. Downgraded since small number of studies included small sample size.

d. Downgraded since there were no significant effects detected.
